# Autism screening and diagnosis in low resource settings: Challenges and opportunities to enhance research and services worldwide

**DOI:** 10.1002/aur.1575

**Published:** 2015-10-06

**Authors:** Maureen S. Durkin, Mayada Elsabbagh, Josephine Barbaro, Melissa Gladstone, Francesca Happe, Rosa A. Hoekstra, Li‐Ching Lee, Alexia Rattazzi, Jennifer Stapel‐Wax, Wendy L. Stone, Helen Tager‐Flusberg, Audrey Thurm, Mark Tomlinson, Andy Shih

**Affiliations:** ^1^ Departments of Population Health Sciences and Pediatricsand Waisman Center, University of Wisconsin‐Madison 789 WARF610 Walnut Street MadisonWisconsin; ^2^ Department of PsychiatryMcGill University1033 Pine Avenue WestMontrealQuebecH3A 1A1Canada; ^3^ Olga Tennison Autism Research CentreSchool of Psychology and Public Health, College of Science, Health, and Engineering, La Trobe UniversityMelbourneVictoria3086Australia; ^4^ Department of Women and Children's HealthInstitute of Translational Medicine, University of Liverpool, Alder Hey Children's NHS Foundation TrustEaton RoadLiverpoolUnited Kingdom; ^5^ MRC SocialGenetic & Developmental Psychiatry Centre, Institute of Psychiatry, Psychology & Neuroscience (PO 80), King's College LondonDe Crespigny ParkDenmark HillLondonUnited Kingdom; ^6^ Department of Life Health & Chemical SciencesFaculty of Science, The Open UniversityWalton HallMilton KeynesUnited Kingdom; ^7^ Department of PsychologyInstitute of Psychiatry, Psychology & Neuroscience, King's College London, Addison House, Guy's CampusLondonSE1 1UL; ^8^ Department of EpidemiologyJohns Hopkins Bloomberg School of Public Health615 N. Wolfe St., SuiteBaltimoreMaryland; ^9^ PANAACEA (Programa Argentino para Niños, Adolescentes y Adultos con Condiciones del Espectro Autista)Repetto 1145Buenos Aires1640Argentina; ^10^ Division of Autism and Related DisordersDepartment of Pediatrics, Emory University School of Medicine, Director, Infant Toddler Clinical Research Operations Marcus Autism, Center, Children's Healthcare of Atlanta1920 Briarcliff RoadAtlantaGeorgia; ^11^ Department of PsychologyUniversity of WashingtonCHDD Box 357920SeattleWashington; ^12^ Department of Psychological & Brain SciencesBoston University100 Cummington MallBostonMassachusetts; ^13^ Pediatrics and Developmental NeuroscienceIntramural Research Program, National Institute of Mental Health 10 Center Dr. Rm 1C250BethesdaMaryland; ^14^ Department of PsychologyStellenbosch UniversityWilcocks BuildingRyneveld StreetStellenboschSouth Africa; ^15^ Autism Speaks1 East 33rd Street, 4th FloorNew York

**Keywords:** diagnosis, early detection, epidemiology, intervention

## Abstract

Most research into the epidemiology, etiology, clinical manifestations, diagnosis and treatment of autism is based on studies in high income countries. Moreover, within high income countries, individuals of high socioeconomic status are disproportionately represented among participants in autism research. Corresponding disparities in access to autism screening, diagnosis, and treatment exist globally. One of the barriers perpetuating this imbalance is the high cost of proprietary tools for diagnosing autism and for delivering evidence‐based therapies. Another barrier is the high cost of training of professionals and para‐professionals to use the tools. Open‐source and open access models provide a way to facilitate global collaboration and training. Using these models and technologies, the autism scientific community and clinicians worldwide should be able to work more effectively and efficiently than they have to date to address the global imbalance in autism knowledge and at the same time advance our understanding of autism and our ability to deliver cost‐effective services to everyone in need. ***Autism Res*** *2015, 8: 473–476*. © 2015 International Society for Autism Research, Wiley Periodicals, Inc.

The autism research and clinical communities today face a global imbalance in our knowledge of autism and in the delivery of services for individuals with autism. Most research into the epidemiology, etiology, clinical manifestations, diagnosis and treatment of autism is based on research in high income countries, even though less than 20% of the world's current population live and fewer than 10% of all births occur in these countries [World Bank, 2015; Population Reference Bureau, 2015]. A recent review of the global prevalence of autism showed that 86.5% of cases identified in epidemiologic studies were from North America, Europe and Japan, though only 10% of the world's children live in these areas [Elsabbagh et al., 2012; Population Reference Bureau, 2015]. Similar imbalances are evident in the global literature on the genetic bases of autism, the contribution of environmental risk factors, the clinical course of autism spectrum disorder, the development and validation of tools for screening and diagnosis, and the design and evaluation of therapies. Thus, even though the vast majority of individuals with autism in the world today live in low and middle income countries, these countries have been largely unrepresented in autism research. The imbalance in knowledge and services also plays out *within* some countries in the form of socioeconomic disparities. As early as 1980, Lorna Wing pointed out that autism is likely to occur with equal frequency among children of different social classes, but that access to an autism diagnosis was preferentially available to children of highly educated parents [Wing, 1980]. This inequity, combined with the voluntary, convenience sampling approaches used for most research projects, including those focused on developing diagnostic tools, has led to the current situation where most autism research and the tools developed for autism assessment and diagnosis are based on studies of highly educated, predominantly white, English speaking populations [Henrich, Heine, & Norenzayan, 2010].

Geographic and socioeconomic disparities in autism research are problematic for two reasons. One is that our knowledge of autism is incomplete and may be biased: because of the concentration of research in high income settings and among white participants, we might miss important cultural, social and biological variability, including variability in the genetic etiology and natural history of autism as well as the potential contribution of environmental exposures that vary globally [Hilton et al., 2010]. The second reason is that research is often linked to capacity‐building through the training of professionals and mid‐level providers and the development of appropriate diagnostic tools and evidence‐based therapies [Collins et al., 2011]. The applicability of existing tools and therapies across cultures and social classes is largely untested and unknown. The paucity of autism research in low resource settings therefore likely contributes to inequities across the world in the ability of individuals with autism and their families to obtain timely access to necessary services and supports. Thus, while the global inequity in autism research and knowledge is a concern in itself, an even greater concern is the likelihood that undeveloped research capacity in low and middle income countries is preventing most of the world's population from benefiting from autism research.

Among the factors perpetuating the imbalance in knowledge and inequity in services is the fact that existing “gold standard” diagnostic tools are extremely difficult for researchers and professionals in low resources countries to access. For example, the most widely used tools, the Autism Diagnostic Interview‐Revised (ADI‐R) and Autism Diagnostic Observation Scale (ADOS), second edition [Lord et al., 2012; Rutter et al., 2003] are costly, require extensive training, and are lengthy to administer. Such tools were developed to fill the need for valid and reliable diagnostic measures to advance research and appropriately directed services. However, the fulfillment of these purposes of autism diagnostic instruments has been thwarted in low resource countries due to barriers related to cost and feasibility. At the same time, because of the extensive available evidence of their validity and reliability, the use of these tools is often required by research funding agencies and peer‐reviewed journals. Autism diagnostic tools are also increasingly used in community based service delivery as a component of diagnostic assessment. “Per use fees” and the inability of users to adapt or translate these tools for use in non‐English speaking settings without paying additional fees, make them inaccessible to most of the world's population. This is true not only for low and middle income countries, but also for low income communities in the United States. This has created an unsustainable situation globally, as the cost of using these tools exceeds per capita annual healthcare expenditures for most of the world's children by more than four‐fold [World Health Organization, 2014]. In addition, for clinicians working in diverse cultural and linguistic environments, it is essential to be able to freely adapt, translate and validate diagnostic tools as needed for use in different settings; however, this practice is prohibited by the licensing restrictions on the most widely used tools.

The development of “gold standards” for autism assessment was initially intended to provide comparability across research samples and to support evidence‐based practice. Indeed, it was in part the availability of the ADI‐R and ADOS as diagnostic instruments that influenced funding agencies to significantly increase their investment in autism research. Over time, the current situation of inequity evolved as a result of several factors. First, at the time these tools were developed in the last century, autism prevalence was thought to be much lower than it is today. With increasing awareness of the condition and recognition of the global impact of the condition, issues of equity and scalability in autism identification have increasingly come to the forefront. Second, advances in research have underscored the heterogeneity of the condition, giving rise to the challenge of developing tools with appropriate sensitivity and specificity to capture the full spectrum. Finally, efforts to improve the situation have been hampered by the use of copyright procedures that were standard practice and perhaps the only available option during most of the 20th Century. Fortunately, the 21^st^ Century has brought new ways of operating with the open source [The Open Source Initiative, 2015] and open access [Suber, 2012] movements, along with advances in internet‐based communication capabilities and models for making essential therapies accessible to patients in low resource settings [Richardson, Grant, & Zolopa, 2014].

What is the way forward? Within the field of software development, “open source” refers to the licensing of intellectual property in a manner that makes products available without cost for anyone to use, modify and share with others. Examples of popular open source or free software and other products include the *Linux* operating system, the *Mozilla Firefox* browser, the user‐generated online encyclopedia *Wikipedia* and the statistical software known as *R* or *The R Project for Statistical Computing*. *R*, for example, is free software made available under a GNU General Public License, which allows the free use, copying, modification and distribution of the software, and also encourages users to work cooperatively to make improvements, develop new programs or modules, and make their products available to the user community [Torsten & Everitt, 2014]. Hallmarks of the open source movement are its reliance on the cooperation and collaboration of many different people around the globe to write and edit code or other materials, and its mission to create high quality products that are more flexible, trusted and/or accessible than proprietary alternatives. Another initiative that shares these characteristics is the highly respected *Cochrane* syntheses of evidence for health and healthcare decision‐making. These initiatives have all required funding and other forms of support for their development and maintenance, but still have as a central principle free access to the resulting information and tools.

Although the tasks involved in developing tools for autism diagnosis may seem to have little in common with software development, the success of the open source movement and its emphasis on global cooperation, high quality, and the freedom to use, adapt and share intellectual property, make it a useful and compelling model for the future development of globally accessible and applicable autism diagnostic tools. Knowledge about autism, like software, can be seen as a “global public good” [Barrett, 2007]. The fact that someone makes use of this knowledge does not prevent others from using it. As with other global public goods, such as the eradication of smallpox and the development vaccines to prevent polio [Barrett, 2007], autism knowledge should benefit all citizens of the world. The adoption of open source principles could potentially allow everyone to access, use and share autism research and tools. At the same time, it should be noted that open source principles do not preclude commercial use or the ability of individuals to earn income from their expertise in the development and use of products based on open source code. For example, the General Public License allows developers and users of a product to profit from distributing it or providing training in its use, but at the same time protects the freedom of others to use, distribute and modify the product as they wish [Gay, 2002].

Models that are related to the open source approach and likely to be just as useful to the autism research community include open access publishing [Suber, 2012] and massive open online courses or MOOCs. Open access copyrights allow publications to be freely accessible, reproducible and distributed while at the same time allowing authors control over the integrity of their work and the right to be properly acknowledged and cited. Information on existing disability screening and assessment tools that are free and openly accessible can be found on the *DisabilityMeasures* website [Maenner, 2015]. The adoption of an open access approach to publishing high quality tools for autism assessment would go a long way toward extending autism research and therapies globally, and addressing the gaps in knowledge and inequities that exist today.

We are, therefore, calling on the autism research community to draw on lessons from the open source movement and to begin to work together to develop, publish and support the use of open access tools for autism assessment, diagnosis, therapy, and research. We see this as the beginning of a broader effort that would not stop at the development of culturally appropriate, reliable and valid tools to document impairments necessary to make a diagnosis. Just as important as having such tools, and perhaps more challenging, is training of professionals and para‐professionals in the diagnosis and treatment of autism and other developmental disabilities, so they can select and make use of a range of culturally appropriate and accessible tools. The diagnosis of autism and other developmental disorders requires clinicians to go beyond assessment of symptoms and to consider daily living tasks and community participation across the lifespan [Baumgartner & Susser, 2012; World Health Organization, 2001]. This will, of course, require continued efforts on the part of autism researchers to establish and maintain high quality in training and use of diagnostic tools, while at the same time addressing the challenges of cross‐cultural comparability and limited resources. Excellent models of open, accessible and high quality materials for training include the International Association for Child and Adolescent Psychiatry and Allied Professionals' online *Textbook of Child and Adolescent Mental Health* and accompanying presentations published under the Creative Commons Attribution Non‐commercial License [Rey, 2015], and The Open University's *Non‐Communicable Diseases, Emergency Care and Mental Health Module* [The Open University, 2015]. Access to these resources requires access to high‐speed internet services, which is still limited for populations in many low income countries. Fortunately, internet access is becoming increasingly available worldwide through mobile technologies. According to a recent report of the London‐based organization GSMA, “By 2020, there will be 6 billion smartphone users worldwide, 80% of whom will be from the developing world” [GSMA, 2015].

In summary, by adopting principles and strategies of open source collaboration, open access publishing, open online learning and related approaches, the autism scientific community should be able to work more effectively than it has to date to address its global imbalance and at the same time advance our understanding of autism across cultures and our ability to deliver cost‐effective services wherever they are needed.
